# Plague: A Millenary Infectious Disease Reemerging in the XXI Century

**DOI:** 10.1155/2017/5696542

**Published:** 2017-08-20

**Authors:** A. J. dos Santos Grácio, Maria Amélia A. Grácio

**Affiliations:** Instituto de Higiene e Medicina Tropical, Universidade Nova de Lisboa, Rua da Junqueira 100, 1349-008 Lisboa, Portugal

## Abstract

Plague, in the Middle Ages known as Black Death, continues to occur at permanent foci in many countries, in Africa, Asia, South America, and even the USA. During the last years outbreaks were reported from at least 3 geographical areas, in all cases after tens of years without reported cases. The recent human plague outbreaks in Libya and Algeria suggest that climatic and other environmental changes in Northern Africa may be favourable for* Y. pestis* epidemiologic cycle. If so, other Northern Africa countries with plague foci also may be at risk for outbreaks in the near future. It is important to remember that the danger of plague reoccurrence is not limited to the known natural foci, for example, those of Algeria, Angola, and Madagascar. In a general context, it is important that governments know the dangerous impact that this disease may have and that the health and medical community be familiar with the epidemiology, symptoms, treatment, and control of plague, so an appropriated and timely response can be delivered should the worst case happen. Plague can be used as a potential agent of bioterrorism. We have concluded that plague is without a doubt a reemerging infectious disease.

## 1. Introduction

Plague, in the Middle Ages known as Black Death, continues to occur at permanent foci in many countries, in Africa, Asia, South America, and even the USA. Its etiologic agent is a bacterium, named* Yersinia pestis (*=*Pasteurella pestis)*. These bacteria live in rats and other rodents, causing among them highly lethal epidemics. When the infected* Xenopsylla cheopis*, the rat flea, leaves the dead body of its rodent host, it goes on to bite any other suitable host, including humans. The flea bitten infected humans fall ill with the so-called bubonic plague. If bubonic plague evolves into a systemic septicaemia, lungs may be affected and a pneumonic plague develops. Both plague septicaemia and pneumonia have a very high mortality. As any other pneumonia, pneumonic plague may be infectious by air drops, without flea transmission. As yet three world pandemics occurred. The first was in the sixth century. It began in Egypt and spread to Europe and Asia Minor, causing 40 million deaths. The second pandemic began in Central Asia in the early 14th century, caused severe epidemic in China and India, moved along caravan routes to Constantinople, and spread through Persia and Middle East to the Mediterranean region. Entering Sicily at Messina in 1347, it swept across Europa and the British Isles in successive waves over the next three centuries. Known as the “Black Death,” medieval plague killed as many as a quarter or more of the affected populations. Third pandemic arose in southern China in the latter half of the nineteenth century, struck Hong Kong in 1894, and over the next several years spread by rat-infested steamships to major port cities throughout the world. Within 35 years of its appearance in Hong Kong the third pandemic had resulted in an estimated 26 million plague cases and more than 12 million deaths; the vast majority was in India [1]. The objective of this work is to review the current knowledge on this infectious disease and to alert the scientific community, doctors, and health service workers and public, in general, and give our evidence of two human plague outbreaks which have occurred in Angola, from November 1973 to March 1974 and from January to March 1975.

## 2. Material and Methods

The elaboration of this work was based on a literature review based on PubMed, Medline Science Direct, and Google Scholar databases, of English, French, and Portuguese articles published between 1970 and 2017, using the terms bubonic plague, bioterrorism, Europe, America, Africa, Middle East,* Yersinia pestis*, and overview history, in relevant references cited in those articles, and our evidence of two human plague outbreaks which have occurred in Angola, from November 1973 to March 1974 and from January to March 1975.

## 3. Results and Discussion

### 3.1. General Review

WHO reported between 1989 and 2003 a total of 38,310 notified human plague cases, with 2,845 deaths, from 25 countries, with the highest number of human plague cases being notified in 1991 and the lowest number being reported in 1989. On the other hand, during the last years outbreaks were reported from at least three geographical areas, in all cases after tens of years without reported cases: India, 1994 and 2002; Indonesia, 1997; Algeria, 2003, in this case, 50 years after its last occurrence, together with the probable discovery of a previously unknown natural focus [2]. In view of these facts we present then a review of the situation of the plague in a global level with basis in a chronologic sequence of data published annually, looking to give to the reader a view of constant presence of plague in the world and of its present expansion. Concerning human plague during 1989–2003 [2], world total of cases (and deaths) reported was 876 (103) in 1989; 1426 (141) in 1990; 3059 (178) in 1991; 2232 (217) in 1992; 2236 (191) in 1993; 2939 (212) in 1994; 2861 (137) in 1995; 3017 (205) in 1996; 5419 (274) in 1997; 2464 (209) in 1998; 2603 (212) in 1999; 2513 (232) in 2000; 2617 (175) in 2001; 1925 (177) in 2002; and 2118 (182) in 2003. These cases were reported from (1)* Africa: Algeria*, with 0 cases from 1989 to 2002 and 11 cases in 2003;* Botswana*, with 103 cases (9 deaths) in 1989 and 70 cases (3 deaths) in 1990 and 0 cases from 1991 to 2003;* Democratic Republic of Congo*, with 289 cases (28 deaths) in 1991, 390 (140 deaths) in 1992, 636 cases (89 deaths) in 1993, 82 cases (10 deaths) in 1994, 582 cases (23 deaths) in 1995, 0 cases in 1996 and 1997, 95 cases (42 deaths) in 1998, 90 cases (29 deaths) in 1999, 371 cases (63 deaths) in 2000, 509 cases (52 deaths) in 2001, 798 (72 deaths) in 2002, and 1092 (68 deaths) in 2003;* Kenya*, with 0 cases in 1989, 44 cases (8 deaths) in 1990, 0 cases from 1991 to 2003;* Madagascar*, with 170 cases (41 deaths) in 1989, 226 cases (55 deaths) in 1990, 137 cases (30 deaths) in 1991, 198 cases (26 deaths) in 1992, 147 cases (23 deaths) in 1993, 126 cases (15 deaths) in 1994, 1147 cases (16 deaths) in 1995, 1629 cases (109 deaths) in 1996, 2863 cases (176 deaths) in 1997, 1473 cases (113 deaths) in 1998, 1304 cases (132 deaths) in 1999, 1333 cases (113 deaths) in 2000, 804 cases (66 deaths) in 2001, 658 cases (96 deaths) in 2002, and 933 cases (109 deaths) in 2003;* Malawi*, with 0 cases from 1989 to 1993, 9 cases (0 deaths) in 1994, 0 cases in 1995 and 1996, 582 cases (11 deaths) in 1997, 0 cases in 1998, 74 cases (4 deaths) in 1999, 0 cases in 2000 and 2001, 242 cases (1 death) in 2002, and 0 cases in 2003;* Mozambique*, with 0 cases from 1989 to 1993, 216 cases (3 deaths) in 1994, 0 cases in 1995 and 1996, 825 cases (18 deaths) in 1997, 430 cases (4 deaths) in 1998, 316 cases (3 deaths) in 1999, 45 cases (0 deaths) in 2002, and 31 cases (0 deaths) in 2003;* Namibia*, with 116 cases (0 deaths) in 1989, 169 cases (10 deaths) in 1990, 1092 cases (45 deaths) in 1991, 458 cases (13 deaths) in 1992, 42 cases (1 death) in 1993, 4 cases (0 deaths) in 1994, 0 cases from 1995 to 1998, 131 cases (11 deaths) in 1999, and 0 cases from 2000 to 2003;* Uganda*, with 0 cases from 1989 to 1992, 167 cases (18 deaths) in 1993, 0 cases from 1994 to 1997, 49 cases (16 deaths) in 1998, 0 cases in 1999, 202 cases (50 deaths) in 2000, 319 cases (42 deaths) in 2001, 60 cases (2 deaths) in 2002, and 24 cases (2 deaths) in 2003;* United Republic of Tanzania*, with 31 cases (4 deaths) in 1989, 364 cases (32 deaths) in 1990, 1293 cases (60 deaths) in 1991, 16 cases (2 deaths) in 1992, 18 cases (0 deaths) in 1993, 444 cases (50 deaths) in 1994, 831 cases (74 deaths) in 1995, 947 cases (64 deaths) in 1996, 504 cases (28 deaths) in 1997, 286 cases (3 deaths) in 1998, 420 cases (15 deaths) in 1999, 74 cases (1 death) in 2000, 2 cases (2 deaths) in 2001, 19 cases (0 deaths) in 2002, and 0 cases in 2003;* Zambia*, with 0 cases from 1989 to 1996, 319 cases (26 deaths) in 1997, 0 cases from 1998 to 2000, 850 cases (3 deaths) in 2001, and 0 cases in 2002 and 2003;* Zimbabwe*, with 0 cases from 1989 to 1993, 392 cases (28 deaths) in 1994, 0 cases in 1995 and 1996, 8 cases (2 deaths) in 1997, 8 cases (2 deaths) in 1998, 9 cases (2 deaths) in 1999, and 0 cases from 2000 to 2003; (2)* Americas: Bolivia*, with 0 cases in 1989, 10 cases (2 deaths) in 1990, 0 cases from 1991 to 1995, 26 cases (4 deaths) in 1996, and 0 cases from 1997 to 2003;* Brazil*, with 26 cases (0 deaths) in 1989, 18 cases (0 deaths) in 1990, 10 cases (0 deaths) in 1991, 25 cases (0 deaths) in 1992, 0 cases in 1993, 4 cases (0 deaths) in 1994, 9 cases (0 deaths) in 1995, 1 case (0 deaths) in 1996, 0 cases (0 deaths) in 1997, 4 cases (0 deaths) in 1998, 6 cases (0 deaths) in 1999, 2 cases (0 deaths) in 2000, and 0 cases (0 deaths) from 2001 to 2003;* Ecuador*, with 0 cases from 1989 to 1997, 14 cases (14 deaths) in 1998, and 0 cases from 1999 to 2003;* Peru*, with 0 cases in 1989, 18 cases (4 deaths) in 1990, 0 cases in 1991, 120 cases (4 deaths) in 1992, 611 cases (31 deaths) in 1993, 420 cases (19 deaths) in 1994, 97 cases (2 deaths) in 1995, 23 cases (0 deaths) in 1996, 39 cases (0 deaths) in 1997, 1 case (0 deaths) in 1998, 22 cases (0 deaths) in 1999, 17 cases (0 deaths) in 2000, 10 cases (0 deaths) in 2001, 2 cases (0 deaths) in 2002, and 0 cases in 2003;* United States of America (USA)*, with 4 cases (0 deaths) in 1989, 2 cases (0 deaths) in 1990, 11 cases (0 deaths) in 1991, 13 cases (2 deaths) in 1992, 10 cases (1 death) in 1993, 14 cases (2 deaths) in 1994, 9 cases (1 death) in 1995, 5 cases (2 deaths) in 1996, 4 cases (1 death) in 1997, 9 cases (0 deaths) in 1998, 9 cases (1 death) in 1999, 6 cases (0 deaths) in 2000, 2 cases (0 deaths) in 2001, 2 cases (0 deaths) in 2002, and 1 case (0 deaths) in 2003 (additional data: according to CDC, in USA, from 1990 to 2010, 999 confirmed or probable cases of plague were reported, being bubonic form in more than 80% of them) [3]; (3)* Asia: China*, with 10 cases (6 deaths) in 1989, 75 cases (2 deaths) in 1990, 29 cases (11 deaths) in 1991, 35 cases (6 deaths) in 1992, 13 cases (1 death) in 1993, 7 cases (4 deaths) in 1994, 8 cases (0 deaths) in 1995, 98 cases (7 deaths) in 1996, 43 cases (0 deaths) in 1997, 0 cases in 1998, 16 cases (5 deaths) in 1999, 25 cases (2 deaths) in 2000, 79 cases (7 deaths) in 2001, 68 cases (0 deaths) in 2002, and 13 cases (1 death) in 2003;* India*, with 0 cases from 1989 to 1993, 876 cases (54 deaths) in 1994, 0 cases from 1995 to 2001, 16 cases (4 deaths) in 2002, and 0 cases in 2003;* Indonesia*, with 0 cases from 1989 to 1996, 6 cases (0 deaths) in 1997, and 0 cases from 1998 to 2003;* Kazakhstan*, with 2 cases (1 death) in 1989, 4 cases (2 deaths) in 1990, 1 case (0 deaths) in 1991, 0 cases in 1992, 3 cases (1 death) in 1993, 0 cases from 1994 to 1996, 1 case (0 deaths) in 1997, 0 cases in 1998, 7 cases (2 deaths) in 1999, 0 cases in 2000, 2 cases (1 death) in 2001, and 0 cases in 2002 and 2003;* Lao People's Democratic Republic*, with data not available from 1989 to 1994, 7 cases (0 deaths) in 1995, 3 cases (0 deaths) in 1996, and 0 cases from 1997 to 2003;* Mongolia*, with 5 cases (3 deaths) in 1989, 15 cases (3 deaths) in 1990, 3 cases (0 deaths) in 1991, 12 cases (4 deaths) in 1992, 21 cases (7 deaths) in 1993, 0 cases in 1994, 1 case (1 death) in 1995, 6 cases (0 deaths) in 1996, 4 cases (2 deaths) in 1997, 10 cases (6 deaths) in 1998, 4 cases (2 deaths) in 1999, 10 cases (3 deaths) in 2000, 8 cases (2 deaths) in 2001, 6 cases (2 deaths) in 2002, and 10 cases (1 death) in 2003;* Myanmar*, with 34 cases (2 deaths) in 1998, 6 cases (0 deaths) in 1990, 100 cases (1 death) in 1991, 528 cases (3 deaths) in 1992, 87 (data not available) in 1993, 6 cases (0 deaths) in 1994, and 0 cases (0 deaths) from 1995 to 2003;* Vietnam*, with 374 cases (37 deaths) in 1989, 405 cases (20 deaths) in 1990, 94 cases (3 deaths) in 1991, 437 cases (17 deaths) in 1992, 481 cases (19 deaths) in 1993, 339 cases (27 deaths) in 1994, 170 cases (10 deaths) in 1995, 279 cases (19 deaths) in 1996, 210 cases (10 deaths) in 1997, 85 cases (7 deaths) in 1998, 195 cases (6 deaths) in 1999, 22 cases (data not available) in 2000, 13 cases (data not available) in 2001, 8 cases (0 deaths) in 2002, and 0 cases in 2003. These global data show an increased incidence of human plague, which was particularly apparent in Africa. This may be associated with both an actual increase in plague activity in its natural foci and an improvement of notification to WHO by Member Status [2].

As regards Angola, West Central Africa, this country is not included in the data reported by WHO [2]. Possibly, the political situation in Angola (including armed conflicts, during several years) impeded the obtainment of data on plague. However, plague is an old problem in Angola. So, the first reference to the presence of plague was done in 1921. Plague outbreaks in Angola were reported from 1932 to 1937, in 1950, 1960, and 1961. After more than a 20 years' gap, two large bubonic plague outbreaks were recorded in 1973-1974 and 1975. The first, from November 1973 to March 1974, occurred in the area of Bocoio/Caimbambo (Benguela District) and it occurred at the same area and the same months of the year as the 1933-1934 outbreak. It is interesting to note the local rural population believed the disease outbreaks had occurred regularly at about every five years [4]. The second outbreak occurred from January to March 1975 at the area of Cuito/Cuanavale and was the first recorded there [4], perhaps a sign of continuous plague activity, underground. The occurrence of a new plague outbreak in 1980-81 at Bocoio (27 cases, 4 deaths, the first time the newly independent country of Angola notified a plague outbreak [5]) seems to confirm the population belief of human plague outbreaks in this area at about every five years [4].

Another theme deserving attention is the occurrence of plague outbreaks in countries where no cases were reported during many years. Here we present some examples. Saudi Arabia had no reported human plague cases for more than 40 years; however in 1994 a new human outbreak was reported [6]. Also, after 70 years with no reported cases, a human outbreak was reported in Jordan [7]. Another case was Algeria, where an outbreak of human plague was reported in June-July 2003, after more than 50 years with no disease activity reported [8]. In 2009, Libya reported 5 confirmed cases, after 25 years of no plague detected [9]. More recently, only from the year 2013, Madagascar reported 84 known plague cases with 42 deaths, from 4 of the country's 112 districts [10]. In New Mexico, there was one human plague case who was hospitalized in critical condition in April 2014, another hospitalized in July 2014, four human plague cases in 2013 with one fatality, one human plague case in 2012, two human cases of plague in 2011, no cases in 2010, and six human cases of plague in 2009, one of them fatal [11–13]. In all countries where it is endemic, plague has showed seasonal variations. Often, these variations can be correlated with rodent fertility and density, its flea's density, and the degree of human proximity to infected animals foci [14]. The recent human plague outbreaks in Libya and Algeria suggest climatic and other environmental changes in Northern Africa may be favourable for* Y. pestis* epidemiologic cycle. If so, other Northern Africa countries with plague foci also may be at risk for outbreaks in the near future [9]. It is important to remember that the danger of plague reoccurrence is not limited to the known natural foci, for example, those of Algeria [8] and of Angola, with two large bubonic plague outbreaks recorded in 1973 and 1975 [4]. In New Mexico, four human plague cases were confirmed in 2015 [15] by the New Mexico Department of Health, in a 73-year-old woman from Santa Fe County. The woman was hospitalized and is back home recovering. The other cases were a 52-year-old woman from Santa Fe County, who died from the illness, and a 65-year-old man and a 59-year-old woman, both from Bernalillo County, who have recovered. Also in New Mexico, in 2016, four more human plague cases were found, in a 16-year-old boy from Rio Arriba County, a 77-year-old man from Bernalillo County, a 21-year-old man from Mora County, and a 67-year-old man from Bernalillo County, who was exposed to plague while in Santa Fe County [16, 17]. In an epidemic hazard in Russia (Asia) on July 2016 it is reported that a ten-year-old boy in Altai Republic (Siberia) has contracted bubonic plague. All 17 people, including 6 children with whom the boy had contact, have been quarantined. It is possible that the boy was infected during a trip to the mountains [18]. More recently, according to WHO [19] new outbreaks have occurred in Madagascar. In fact, in disease outbreaks news of 9 January 2017 it is referred: “On 6 December 2016, the Ministry of Health (MoH) in Madagascar alerted WHO of a suspected plague outbreak in Befotaka district, Atsimo-Atsinanana region in the south-eastern part of the country. The district is outside the area known to be endemic area in Madagascar. No plague cases have been reported in this area since 1950. As of 27 December 2016, 62 cases (6 confirmed, 5 probable, 51 suspected) including 26 deaths (case fatality rate of 42%) have been reported in two adjacent districts in two neighbouring regions of the country. 28 cases, including 10 deaths, have been reported from Befotaka District in Atsimo-Atsinanana Region and 34 cases including 16 deaths have been reported from Iakora district in Ihorombe Region. Of the 11 samples tested, 5 were positive for plague on rapid diagnostic test and 6 are now confirmed at Institute Pasteur laboratory. Of the total reported cases, 5 are classified as pneumonic plague cases and the remaining as bubonic plague. Retrospective investigations carried out in those two districts showed that it is possible that the outbreak might have started in mid-August 2016. The investigation in neighbouring villages is still ongoing. On 29 December, an investigation carried out within 25 km of the initial foci in Befotaka district has reported three deaths and is being investigated further for possible linkage to the outbreak.” Considering that plague may be infectious by air drops (without fleas transmission) it is necessary to alert the health workers for the danger of infection when they are working in areas with that disease. For example, in Portugal, in 1899, Dr. Carlos França and Professor Câmara Pestana have worked on human plague, and they eradicated in Porto City an epidemic of bubonic plague and both of them were infected, but only Professor Câmara Pestana died because of that disease. In homage, Portugal established the “Instituto Bacteriológico Câmara Pestana” [20].

### 3.2. Our Evidence on Two Human Plague Outbreaks in Angola

#### 3.2.1. Introduction

**Figure 1:**
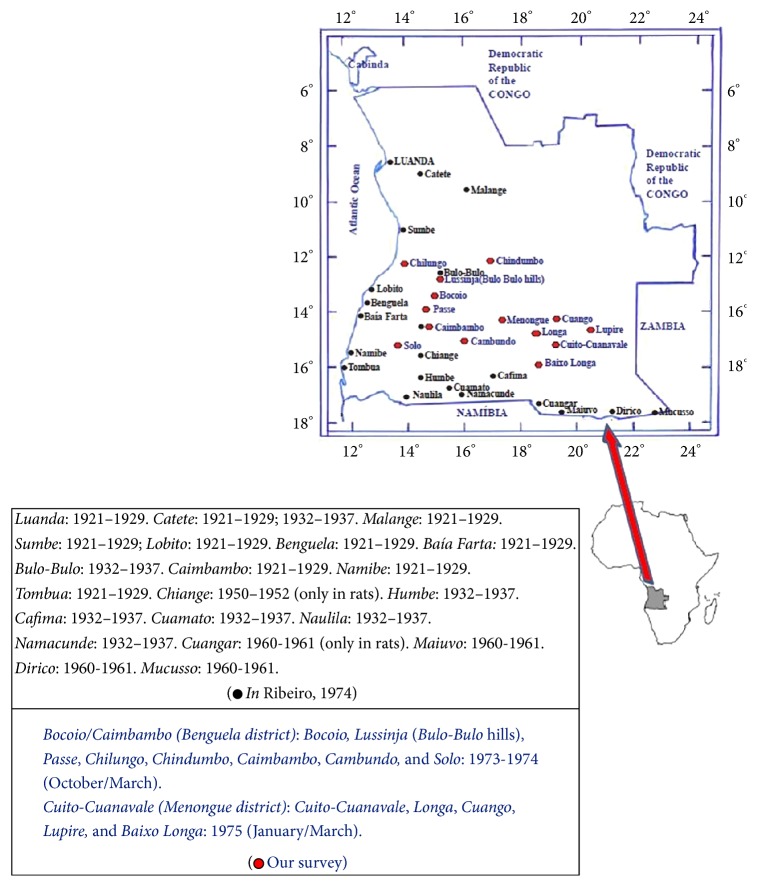
Distribution of human and animal plague in Angola from 1921 to 1981.

Here, our objective is to describe two outbreaks of bubonic plague of 1973-1974 and 1975 and to present a historical overview of the plague in Angola based on our participation in those outbreaks and our paper [4]. In Angola, plague was introduced in 1921 by maritime way in Luanda, where the first epidemic has occurred being also introduced in other ports, namely, Novo Redondo (November, 1921), Lobito (1921–1925), Benguela (1921–1927), Moçamedes (1922), and Porto Alexandre (1922–1929) and, on the other hand, in two localities situated in the interior, Catete, relatively near Luanda (1921–1923), and Malanje (1921–1923) (Figure 1) [4]. In 1932, the sylvatic plague of the Southern Africa reaches the frontiers of the South from Angola. In December 1933 and January–April 1934 plague occurred in the Benguela district areas of Bocoio (Bulo Bulo hills) and Caimbambo and in Catete the disease was again observed in 1933-1934 (Figure 1). In 1934 and 1937 some cases of human plague were recorded in Namacunde and in 1935 in Dombandola (Figure 1). In October 1950 an outbreak of human plague was recorded in Baía Farta (Benguela district). In 1952 an epizootic of the wild rodents (*Tatera schinzi* and others) reaches the suburbs of Vila Pereira d'Eça, but no human cases of plague were recorded. In December 1960 and January 1961, after an epizootic initially, some human cases of bubonic plague were recorded in Baixo Cubango, in Cuangar, Maiuvo (Chimpande), Dirico, and Mucusso (Figure 1). Between November 1973 and March 1975 two outbreaks of bubonic plague have occurred in Angola (Benguela and Menongue districts). In 1980-1981 a new plague outbreak has occurred at Bocoio-Benguela district [5].

#### 3.2.2. Population and Areas Study

Women, men, youths, and children were included in our studies. In the first outbreak of bubonic plague (November 1973–March 1974) they were living in Bocoio, Lussinja (Bulo Bulo hills), Passe, Chilungo, Chindumbo, Caimbambo, Cambundo, and Solo, between 12° 28′S/14° 08′E and 13° 01′S/14° 00′E, all these localities being situated in Benguela district. In the second outbreak (January–March 1975) they were living in the area of Cuito Cuanavale (Menongue district), in the localities of Cuito Cuanavale, Longa, Cuango, Lupire, and Baixo Longa, between 14° 28′S/18° 55′E and 15° 42′S/18° 39′E.

In the first outbreak a total of 839 people were examined and about 200 were hospitalized in the Bocoio Hospital and Passe Sanitary Center. However, when we initiated the works in this outbreak already it was occurring there for at least one month. This outbreak was known in a case of an old man attending Bocoio Hospital when the doctor had indicated to the nurse to lance the boil that he had in inguinal region, and the man said “this is not a boil but a swollen gland of plague which occurs in this area of five an five years.” We say that this outbreak was occurring there for at least one month, one month before two doctors of the Angolan Health Service traveling from Benguela to Luanda have observed several rats dead or running along the road. In that time they had mentioned to us that, in the presence of those facts, they had commented that “this appears as a plague epidemic.” In the second outbreak owing to politic situation, it is not possible to examine and to treat population in the Menongue Hospital. So, groups of nurses and other health staff examined and treated the patients in their homes.

#### 3.2.3. Patient

The individuals presenting fever and lymphadenitis, without another obvious cause of infection, were considered plague cases. Specimens of aspirated buboes, sputum, and blood (in case of hyperthermia) of patients, as well as specimens of spleen, liver, lung, ganglia, and intracardiac blood from human corps were sent to the Bacteriology Laboratory of the “Instituto de Saúde Pública de Angola” for bacterial culture. Once confirming the presence of plague between the populations, the individuals with evidence of typical clinic symptoms were considered as positive, since, according to WHO, that evidence is enough to lead the physicians and nurses to establish with a good margin of safety the diagnosis of plague [21].

#### 3.2.4. Rodents

**Figure 2:**
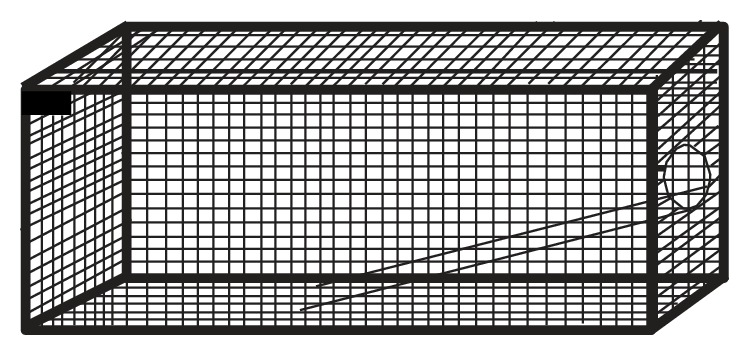
Rodents trap.

Rodents were trapped using closed hundred traps (Figure 2) distributed by field around the homes in the different localities with human cases of plague.

Rodents trapped and found dead were used for obtainment of specimens of liver, spleen, lung, ganglia, and intracardiac blood, for bacterial culture. The isolates of the bacterial cultures were sent for the University of California, San Francisco, USA, for identification confirmation.

#### 3.2.5. Entomological Survey

Fleas were obtained from trapped rodent and domestic and peridomestic animals, principally cats* (Felis catus)* and dogs* (Canis familiaris)*. The fleas identification was made according to De Meillon et al., 1961 [22], and Rothschild and Traub, 1971 [23].

## 4. Results

### 4.1. Outbreak of Bubonic Plague in Benguela District (November 1973–March 1974)

#### 4.1.1. Human Plague

**Figure 3:**
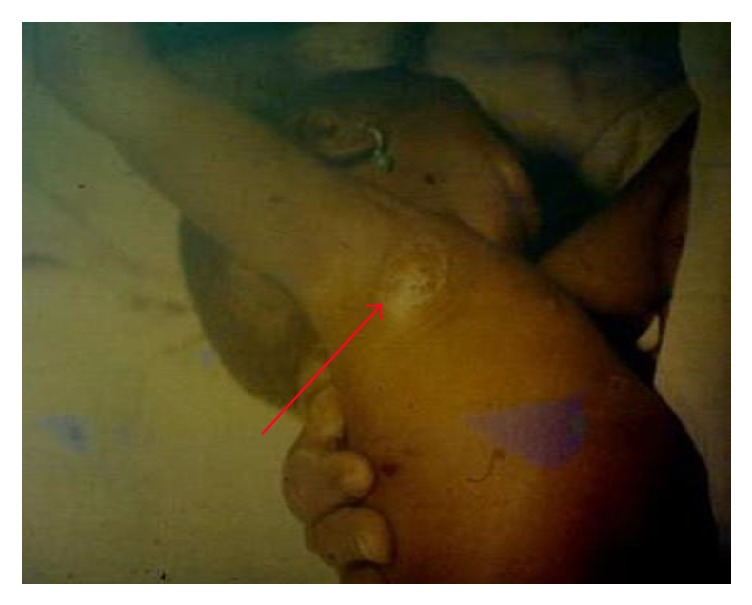
An axillary bubo of a child infected with bubonic plague (photo: A. J. Santos Grácio).

**Figure 4:**
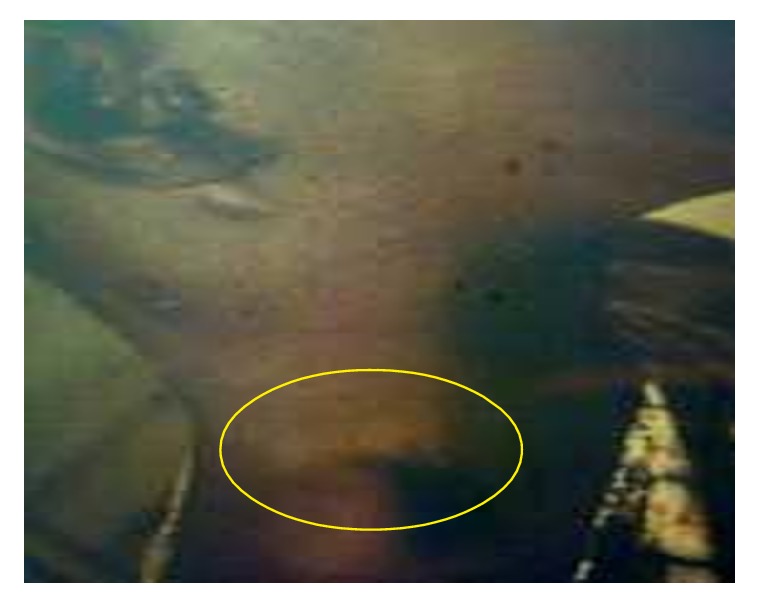
A bubo on the neck of a woman with bubonic plague (photo: A. J. Santos Grácio).

**Figure 5:**
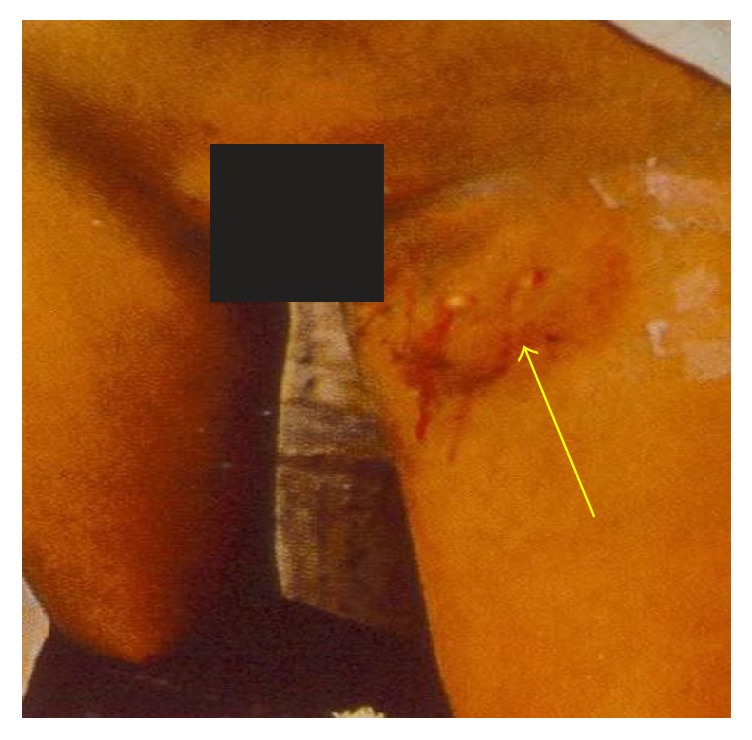
A bubo on the thigh close to inguinal zone of a girl with bubonic plague (photo: A. J. Santos Grácio).

**Figure 6:**
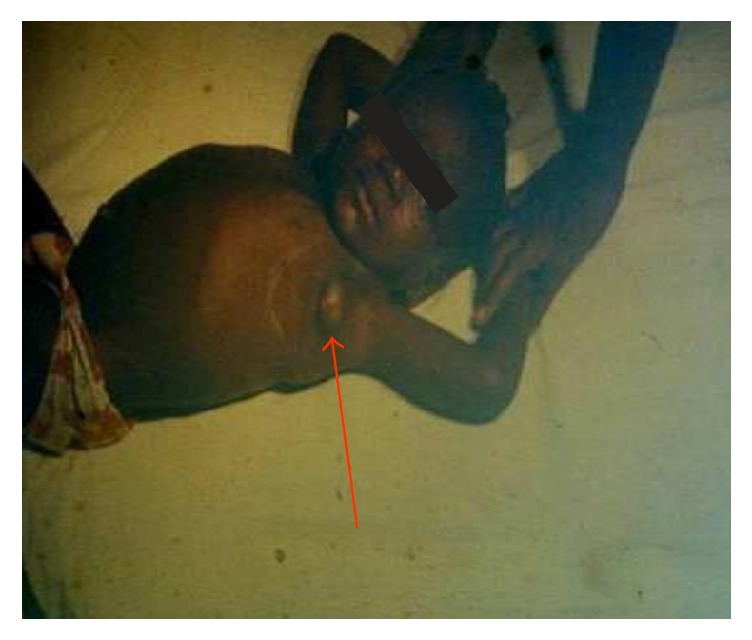
An axillary bubo of a child with bubonic plague (photo: A. J. Santos Grácio).

**Figure 7:**
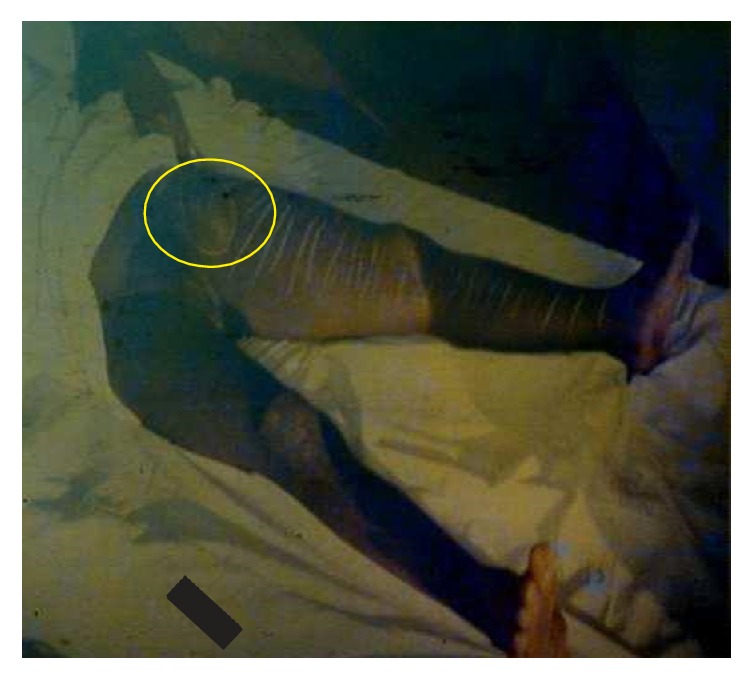
An inguinal bubo of a boy with bubonic plague. The lines show the pain area in the leg (photo: A. J. Santos Grácio).

**Figure 8:**
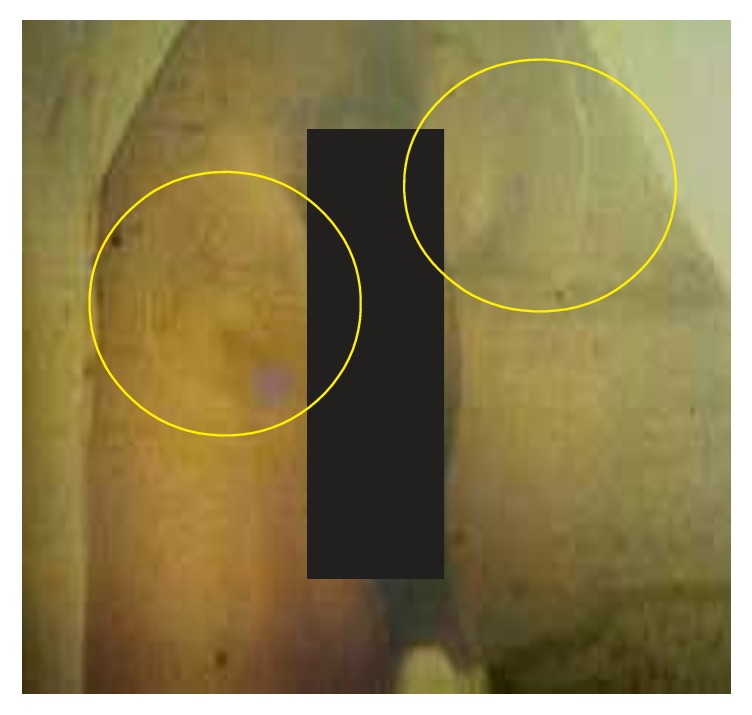
Two inguinal buboes of a man with bubonic plague (photo: A. J. Santos Grácio).

About 200 individuals who were hospitalized in Bocoio Hospital all had buboes situated in different parts of the body (inguinal, axial, and neck areas) (Figures 3, 4, 5, 6, 7, and 8). All individuals were treated and only 11 of them died.

#### 4.1.2. Entomological Survey

Fleas (Siphonaptera) identified

 
*Xenopsylla brasiliensis* (Baker, 1904)
 
*X. cheopis *(Rothshild, 1903)^*∗*^
 
*X. versuta* (Jordan, 1925)
 
*X. phylloxera* Hopkins, 1949
 
*Xenopsylla* sp.
 
*Echidnophaga gallinacea* (Westwood, 1875)

^*∗*^*X*.  *cheopsis* is the principal responsible flea for transmission of the plague; it is the primary vector of* Yersinia pestis.*

#### 4.1.3. Rodents and Other Mammals

The fleas identified during the plague outbreak were captured on the following species:

 
*Pelomys campanae* (Huet, 1888)
 
*Rattus (Mastomys) natalensis* A. Smith, 1834 (Ellerman & Morrison-Scott, 1953)
 
*Rattus norvegicus* (Berkenhout, 1796)
 
*Rattus rattus* Linnaeus, 1758
 
*Tatera schinzi* Noack, 1889
  Others.

Rats were responsible for the spread of the plague, since they die in great number, and fleas were to seek other sources of the food (blood), namely, humans.

### 4.2. Outbreak of Bubonic Plague in Menongue District, Cuito-Cuanavale Area (January–March 1975)

#### 4.2.1. Human Plague

In Menongue district it was not possible to observe and treat all cases of bubonic plague in the local hospital because of the tense political and military situation at the time in Angola. So, after localization of the homes of patients, these were observed, and all the individuals with buboes were treated. It would appear that the outbreak had possibly started one/two months ago, because many deaths were occurring in the population and clinical observations recorded were compatible with those attributed to the bubonic plague.

#### 4.2.2. Entomological Survey

Fleas (Siphonaptera) identified

 
*Xenopsylla brasiliensis* (Baker, 1904)
 
*X. cheopis *(Rothshild, 1903)^*∗*^
 
*X. phylloxera* Hopkins, 1949
 
*X. hipponax* De Meillon, 1942
 
*X. georychi* (C. Fox, 1914)
 
*Xenopsylla* sp.
 
*Echidnophaga gallinacea* (Westwood, 1875)

^*∗*^Primary vector of* Yersinia pestis*.

#### 4.2.3. Rodents and Other Mammals

The fleas identified during the plague outbreak were captured on the following species:

 
*Canis familiaris* (Linnaeus, 1758)
 
*Felis catus* Linnaeus, 1758
 
*Pelomys campanae* (Huet, 1888)
 
*Rattus (Mastomys) natalensis* A. Smith, 1834 (Ellerman & Morrison-Scott, 1953)
 
*Rattus norvegicus* (Berkenhout, 1796)
 
*Rattus rattus* Linnaeus, 1758
 
*Tatera brantsi* (Smith, 1834)
 
*Tatera schinzi* Noack, 1889
  Others.

As regards rats, the situation was similar to that observed in 1973-1974. As regards dogs and cats, they may also bring plague-infected fleas into the home and so they were also responsible for the spread of plague.

## 5. Conclusions

### 5.1. On Angolan Human Outbreak of Plague

Ribeiro et al. [24] carried out a survey from 20th April to 30th May 1963 on systematic and bacteriological situation of 239 rodents and insectivora captured in Benguela district, as well as the identification of their fleas and those taken from local domestics dogs, with the objective of knowing the plague situation in that district, in which areas with plague had been recorded in the past.

The authors have referred that all bacteriological examinations were negative. Then, they concluded that* “the absence of any denunciatory signs of present activity of the surveyed foci is probably due to either their effective extinction or their extremely reduced activity.”* However, the occurrence of the outbreak of bubonic plague in those areas of the Benguela district in 1973-74 shows that the foci were not extinct and had normal activity.

We think that the no correspondence between the conclusions of those authors and the reality of the facts occurring in the foci was due the two limitations of study which were recognized by authors:* “the small number of rodents studied (235)*” and* “the short period with available data (end of rainy season).”*

The fact of the indigenous population of areas of the Benguela district, where we carried out our work mentioning the occurrence of the disease at intervals of five years, is also a sign that the plague had a permanent activity in Benguela district. On the other hand the occurrence of new plague outbreak in 1980-1981 at Bocoio in Benguela district (with 27 cases; 4 deaths), which was the first time that plague had been notified from Angola since 1975 [24], shows that the people had reason to relate that the human plague occurs in that area at intervals of five years. It is also interesting to note that in Benguela district the disease was recorded in the same months of the outbreak of 1933-1934.

For the first time plague was recorded in Cuito-Cuanavale's area (Menongue district).

At present, with the armed conflict in Angola being finished, it should be important to carry out surveys in Benguela district and Cuito-Cuanavale' areas with the objective of knowing the actual situation of those plague foci which were in complete activity and are more than three decades ago and, on the other hand, establishing a plague surveillance programme.

### 5.2. General Conclusions

In conclusion, plague is without doubt a reemerging infectious disease. It is important that governments know the dangerous impact this disease may have. It is also important for the health and medical community to be familiar with the epidemiology, symptoms, diagnosis, treatment, and control of plague, so an appropriated and timely response can be delivered should the worst case happen. The importance of this knowledge can be demonstrated with the outbreak of human pneumonic plague with dog-to-human and possible human-to-human transmission, which has occurred in June-July 2014 in Colorado and where* Yersinia pestis* had been previously misidentified as* Pseudomonas luteola* by an automated system in the laboratory hospital [25].

Also the new outbreaks in Madagascar confirm that human plague can be considered an infectious disease reemerging in XXI Century. On the other hand, in a study concerning perspectives on the spread of the plague in Africa it was concluded that all African countries should be concerned by the possible emergence and reemergence of the disease and that it is crucial to implement some preventive measures in these countries [26].

Here we aim to alert for human plague, an infectious disease that was responsible for three world pandemics (first with 40 million deaths, second killing as many as a quarter or more of the affected population, and third with 26 million cases and 12 million deaths) and that can be used as a potential agent of bioterrorism [27].
